# Propensity score regression analysis of oesophageal adenocarcinoma treatment with surgery alone or neoadjuvant chemotherapy

**DOI:** 10.1002/bjs5.50287

**Published:** 2020-05-06

**Authors:** A. G. M. T. Powell, A. Karran, P. Blake, A. Christian, S. A. Roberts, W. G. Lewis

**Affiliations:** ^1^ Division of Cancer and Genetics Cardiff University, and South‐East Wales Cancer Network Cardiff UK; ^2^ Department of Surgery Cardiff UK; ^3^ Department Pathology Cardiff UK; ^4^ Department Radiology University Hospital of Wales Cardiff UK

## Abstract

**Background:**

Propensity score (PS) regression analysis can be used to minimize differences between cohorts in order to perform comparisons The aim of this study was to use PS analysis to examine the outcomes of oesophageal adenocarcinoma (OAC) treatment with surgery alone or neoadjuvant chemotherapy (NAC) followed by surgery (NACS), to see whether the benefits seen in a randomized trial (MRC OE02) were reproducible in a UK cancer network clinical practice.

**Methods:**

Consecutive patients undergoing potentially curative treatment for OAC in a regional cancer network were studied. Multiple regression models, including PS analysis, were developed to account for confounding factors. Primary outcome measures were disease‐free (DFS) and overall (OS) survival.

**Results:**

A cohort of 440 patients was included in a regression analysis controlling for confounders (176 surgery alone, 264 NACS). NACS was associated with a higher positive margin status rate compared with surgery alone (42·4 *versus* 26·7 per cent respectively; *P* < 0·001), an inferior 5‐year DFS rate (32·1 *versus* 56·9 per cent; *P* < 0·001) and a worse 5‐year OS rate (27·5 *versus* 47·3 per cent; *P* < 0·001). On regression adjustment based on propensity scores, NACS was not associated with DFS (*P* = 0·220) or OS (*P* = 0·431). The Mandard tumour regression grade (TRG) score was significantly associated with DFS (hazard ratio (HR) 0·21, 95 per cent c.i. 0·07 to 0·70) and OS (HR 0·27, 0·13 to 0·59). Five‐year DFS and OS rates related to TRG were 64 and 62 per cent respectively for 25 good responders *versus* 8·0 and 8·6 per cent for 127 poor responders (*P* < 0·001).

**Conclusion:**

The prescription of NAC to all patients with OAC risks delay in effective treatment of patients who are relatively chemoresistant, given the variability in pathological response. Identification of patients with OAC who may derive the most benefit from NAC should be the focus.

## Graphical abstract

Following propensity adjustment for stage, age and sex, neoadjuvant therapy was not associated with survival. Tumour regression is a powerful prognostic indicator, and future research should focus on identifying patients who will derive the most benefit from neoadjuvant chemotherapy.

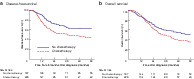

New approaches needed to identify non‐responders to chemotherapy

## Abstract (es)

**Antecedentes:**

El análisis de regresión por puntaje de propensión (*propensity score*, PS) puede ser utilizado para minimizar las diferencias entre cohortes a la hora de hacer comparaciones. El objetivo de este estudio fue utilizar el PS para analizar los resultados del tratamiento del adenocarcinoma de esófago (*oesophageal adenocarcinoma*, OAC), tanto con cirugia sola (*surgery*, S) o con quimioterapia neoadyuvante (*neoadjuvant chemotherapy*, NAC) seguida de cirugía (NACS) para determinar si los beneficios del ensayo aleatorizado MRC OE02 eran reproducibles en la práctica clínica de una red de cáncer del Reino Unido.

**Métodos:**

Se estudiaron pacientes consecutivos sometidos a tratamiento potencialmente curativo por OAC en una red de cáncer regional. Se desarrollaron modelos de regresión múltiple, incluyendo PS, para poder ajustar por factores de confusión. Las medidas de resultado primario fueron supervivencia libre de enfermedad (*disease‐free survival*, DFS) y la supervivencia global (*overall survival*, OS).

**Resultados:**

Se incluyó una cohorte de 440 pacientes en un análisis de regresión controlando por factores de confusión (176 S, 264 NACS). NACS se asoció con margen positivo (NACS *versus* S, 42,4% *versus* 26,7%, *P* < 0,001), menor DFS a los 5 años (32,1% *versus* 56,9, *P* < 0,001) y peor OS a los 5 años (27,5% *versus* 47,3%, *P* < 0,001). En el ajuste de la regresión basada en las puntuaciones de propensión, NACS no se asoció a DFS (*P* = 0,220) ni a OS (*P* = 0,431). El grado de regresión tumoral de Mandard (*tumour regression grade*, TRG) se asoció significativamente con DFS (cociente de riesgos instantáneos, *hazard ra*tio, HR 0,21, i.c. del 95% 0,13‐0,59). La DFS y OS a los 5 años en relación con TRG fue 63,6% y 61,5% *versus* 8,0% y 8,6% (*P* < 0,001) para buenos y pobres respondedores, respectivamente.

**Conclusión:**

La indicación de NAC a todos los pacientes con OAC representa un riesgo de demorar un tratamiento efectivo para aquellos pacientes que son relativamente quimiorresistentes, dada la variabilidad en la respuesta patológica. Identificar a los pacientes con OAC que obtendrían el mayor beneficio de la NAC debería centrar el foco de atención.

## Introduction

The optimal treatment strategy for patients diagnosed with oesophageal adenocarcinoma (OAC) is controversial. As most patients have at least locoregional disease at presentation, multimodal therapy is used widely. UK guidance recommends neoadjuvant chemotherapy (NAC) followed by surgery (NACS)^1^, whereas chemoradiotherapy is more widely used in the neoadjuvant phase in many other European countries^2^. In North America, NAC followed by surgery is often accompanied by adjuvant postoperative chemotherapy or chemoradiotherapy^3^.

RCTs attempting to establish survival benefit for treatment with NACS compared with surgery alone have reported contrasting outcomes. In the two largest of these studies, the UK MRC OE02 trial^4^ reported a 5‐year survival rate of 23·0 per cent after NAC compared with 17·1 per cent after surgery alone (hazard ratio (HR) 0·82, 95 per cent c.i. 0·71 to 0·95; *P* = 0·03), whereas the US RTOG trial 8911 (US Intergroup 113) reported equivalence^5^. A Cochrane review^6^ considered these two studies to be of high quality with low risk of bias, and concluded that, although NACS may offer a survival advantage over surgery alone, further research was required.

Propensity score (PS) analysis is being used increasingly to compare non‐randomized cohorts^7^. It enables estimates of probability of undergoing a treatment given a vector of observed variables and is a powerful alternative for drawing causal inference on observational data compared with conventional case‐mix adjustment. This is based on the adjustment made by PS analysis for confounding factors (or baseline characteristics) on the independent variable (for example treatment option). PSs are generated by a logistic regression model and aim to replace a group of baseline characteristics with one score. Following this, PSs can be used in a number of analytical techniques, the most common being matching, stratification and regression adjustment. In this way, treatment arms can be balanced in terms of important co‐variables, allowing a fair comparison of treatments to be made^8^, ^9^, ^10^, ^11^, ^12^. As much of the selection bias is adjusted for, PS analysis provides a scientifically sound alternative to RCTs in situations where interventions cannot be allocated randomly for ethical and practical reasons^8^, ^9^.

The aim of this study was to examine the outcomes of OAC treatment with surgery alone or with NACS, by means of PS regression analysis, to see whether the benefits suggested in the MRC OE02 trial were reproducible in contemporary clinical practice in a UK regional cancer network.

## Methods

The study included consecutive patients diagnosed with potentially curable oesophageal cancer of adenocarcinoma cell type between 1 January 2003 and 30 June 2018, by a regional multidisciplinary team serving a population of 1·76 million. Clinical and pathological information was collected prospectively. Preoperative staging involved CT, endoluminal ultrasonography (EUS) and laparoscopy, if appropriate. For all patients diagnosed from 2009 onwards, CT–PET has been incorporated routinely. All staging was done in accordance with the UICC TNM seventh edition^13^. Pathological response to chemotherapy was determined using the Mandard tumour regression grade (TRG) score^14^, and was recorded from pathology reports issued at the time of resection. EUS examinations were performed or supervised by one of two radiologists.

Ethical approval was sought from the regional ethics committee, but the chair confirmed that individual patient consent was not required to report clinical outcomes alone and thus no formal approval was necessary.

### Surgery with or without neoadjuvant chemotherapy

Before the publication of the initial OE02 results in 2002^4^, the main curative treatment for these patients was primary surgery. However, after this, fit patients with T3 and equivocal T4, N0 and N1 tumours were generally treated with neoadjuvant therapy before surgery^15^. The majority of these patients received two cycles of cisplatin 80 mg/m^2^ and 5‐fluorouracil (5‐FU) 1000 mg/m^2^ for 4 days. A minority received four cycles of epirubicin 50 mg/m^2^, cisplatin 60 mg/m^2^ and 5‐FU 200 mg/m^2^ or capecitabine 625 mg/m^2^. Other slightly altered regimens were used, depending on patient co‐morbidity or adverse reactions. CT, after the final dose of NAC and before surgery, was used to establish tumour response to NAC^16^.

Patients treated with neoadjuvant chemoradiotherapy were excluded. Patients with radiologically perceived T1–2, N0 disease, and those considered unsuitable to receive chemotherapy because of other co‐morbidities, were offered surgery alone.

Most patients had transthoracic oesophagectomy (TTO) as described by Tanner^17^ and Lewis^18^. Transhiatal oesophagectomy (THO), as described by Orringer^19^, was used selectively in patients with adenocarcinoma of the lower third of the oesophagus who had significant cardiorespiratory co‐morbidity. Some patients with type 2 junctional cancers who underwent an extended total gastrectomy were also included. Oesophageal resection was defined as potentially curative when all visible tumour had been removed. Involvement of the circumferential resection margin was defined as the presence of tumour less than 1 mm from the circumferential margin^20^.

### Follow‐up and disease recurrence

All patients were reviewed every 3 months for the first year after oesophagectomy, and every 6 months thereafter. Disease recurrence was based on clinical suspicion and confirmed by radiological investigation or endoscopy. Patterns of recurrence were defined as locoregional, distant (metastatic), or both locoregional and distant when both were diagnosed at the same time. The time of recurrence was taken as the date of the confirmatory investigation. Death certification was obtained from the Office for National Statistics.

### Statistical analysis

Sample size calculations were based on a prestudy literature survey of Cancer Research UK cancer statistics^21^, which indicated that the baseline 5‐year survival rate in patients diagnosed with stage II OAC was expected to be 40 per cent, compared with 20 per cent in patients with stage III OAC, and that a 15 per cent difference in survival would be a realistic expectation. Thus, a minimum of 276 patients were needed to provide 80 per cent power to detect such a difference with *P* < 0·050.

PSs were generated using a logistic regression model, and included all relevant independent variables thought to be potential confounding factors. These were considered by the regional multidisciplinary team, and comprised patient demographics (age above 70 years and sex) and clinical staging (cTNM) based on radiological assessment of T and N status^22^. Generated PSs were then used in a regression adjustment to estimate the effect of the exposure to treatment on disease‐free (DFS) and overall (OS) survival.

Complete case analysis was based on intention to treat, and the primary outcome measures were DFS and OS. Secondary outcome measures included OAC recurrence and postoperative morbidity. Grouped data were expressed as median (i.q.r.) values, and non‐parametric statistical methods were used. Continuous data were compared using the Mann–Whitney *U* test and categorical data using the χ^2^ test and Fisher's exact test when the number of events was low. DFS for all patients was calculated using methodology similar to that of both the MRC OEO2 and US Intergroup randomized trials, by measuring the period from a landmark time of 6 months after diagnosis to the date of recurrence to allow for the variable interval to surgery after diagnosis, depending on whether NAC was prescribed^4^. As in the above trials, events resulting in a failure to complete curative treatment, such as not proceeding to surgery, open and close laparotomy, palliative resection and in‐hospital mortality, were assumed to have occurred at this landmark time, to maintain the intention‐to‐treat analysis. OS was measured from the date of diagnosis to date of death or censorship, whichever occurred first. Cumulative survival was calculated according to the Kaplan–Meier method, and differences between groups were analysed with the log rank test. Univariable analyses were done initially to examine factors influencing survival, and those with associations found to be statistically significant (*P* < 0·050) were retained in a Cox proportional hazards model. Cox models (controlling for PS) were used to estimate the effect of the treatment on the outcomes, DFS and OS. Data analysis was performed using the SPSS® version 25 (IBM, Armonk, New York, USA).

## Results

Some 440 patients underwent radical treatment with curative intent for OAC, surgery alone in 176 (40·0 per cent) and NACS in 264 (60·0 per cent). Of the 393 patients who had a resection, 188 patients (47·8 per cent) had TTO, 182 (46·3 per cent) had THO, and 23 (5·9 per cent) had an extended total gastrectomy. The other 47 patients (10·7 per cent) had open and close procedures. The median age of patients was 71 (i.q.r. 65–78) years, 368 (83·6 per cent) were men and 72 (16·4 per cent) were women. The overall median lymph node harvest was 14 (i.q.r. 9–20). Some 221 patients (50·2 per cent) developed postoperative morbidity, which was associated with 14 deaths (3·2 per cent) within 30 days of surgery. During follow‐up, 163 patients (37·0 per cent) developed cancer recurrence and 246 (55·9 per cent) died. The median follow‐up of survivors was 60 (range 6–60) months. Some 80·7 per cent of patients were followed up for at least 5 years or until death.

### Variation in clinicopathological factors and perioperative outcomes

Details of 440 patients related to treatment modality are shown in *Table* 1. The operative approach in the surgery‐alone compared with the NACS cohort was TTO in 42 (23·9 per cent) and 146 (55·3 per cent) patients respectively (*P* < 0·001), and THO in 114 (64·8 per cent) and 68 (25·8 per cent) (*P* < 0·001). The rate of open and close laparotomy was nine (5·1 per cent) for the surgery‐alone cohort, compared with 38 (14·4 per cent) for the NACS cohort (*P* = 0·002).

**Table 1:** Relationship between neoadjuvant therapy and clinicopathological factors

	Surgery alone (*n* = 176)	Neoadjuvant chemotherapy + surgery (*n* = 264)	*P* †
**Preoperative factors**			
Age (years)			< 0·001
< 65	31 (17·6)	67 (25·4)	
65–75	64 (36·4)	133 (50·4)	
> 75	81 (46·0)	64 (24·2)	
Sex			0·752
F	30 (17·0)	42 (15·9)	
M	146 (83·0)	222 (84·1)	
ASA grade			0·129
II	76 (43·2)	95 (36·0)	
III	100 (56·8)	169 (64·0)	
Differentiation			0·011
Well/moderate	59 of 92 (64·1)	71 of 150 (47·3)	
Poor	33 of 92 (35·9)	79 of 150 (52·7)	
cT category			< 0·001
cT1	61 (34·7)	4 (1·5)	
cT2	46 (26·1)	35 (13·3)	
cT3	64 (36·4)	197 (74·6)	
cT4	5 (2·8)	28 (10·6)	
cN category			< 0·001
cN0	125 (71·0)	86 (32·6)	
cN1	44 (25·0)	151 (57·2)	
cN2	6 (3·4)	22 (8·3)	
cN3	1 (0·6)	5 (1·9)	
cTNM stage			< 0·001
I	60 (34·1)	2 (0·8)	
IIa	1 (0·6)	2 (0·8)	
IIb	40 (22·7)	19 (7·2)	
III	68 (38·6)	215 (81·4)	
IVa	7 (4·0)	26 (9·8)	
**Perioperative factors**			
Type of surgery			< 0·001
TTO	42 (23·9)	146 (55·3)	
THO	114 (64·8)	68 (25·8)	
Extended gastrectomy	11 (6·3)	12 (4·5)	
Open and close	9 (5·1)	38 (14·4)	
pT category			< 0·001
Complete response	0 (0·0)	10 (3·8)	
pT1	71 (40·3)	18 (6·8)	
pT2	23 (13·1)	22 (8·3)	
pT3	58 (33·0)	156 (59·1)	
pT4	15 (8·5)	20 (7·6)	
No resection	9 (5·1)	38 (14·4)	
pN category			< 0·001
pN0	99 (56·3)	79 (29·9)	
pN1	41 (23·3)	71 (26·9)	
pN2	18 (10·2)	45 (17·0)	
pN3	9 (5·1)	31 (11·7)	
No resection	9 (5·1)	38 (14·4)	
pTNM stage			< 0·001
Complete response	0 (0·0)	11 (4·2)	
I	78 (44·3)	27 (10·2)	
II	36 (20·5)	49 (18·6)	
III	53 (30·1)	139 (52·7)	
IV	0 (0·0)	0 (0·0)	
No resection	9 (5·1)	38 (14·4)	
R status			0·001
R0	120 (68·2)	114 (43·2)	
R1	47 (26·7)	112 (42·4)	
R2	9 (5·1)	38 (14·4)	
Lymph node yield*	12 (8–17)	15 (12–21)	0·001‡
Postoperative morbidity			0·139
No	80 (45·5)	139 (52·7)	
Yes	96 (54·5)	125 (47·3)	
Operative mortality			0·183
No	168 (95·5)	258 (97·7)	
Yes	8 (4·5)	6 (2·3)	

Values in parentheses are percentages unless indicated otherwise;

* values are median (i.q.r.). TTO, transthoracic oesophagectomy; THO, transhiatal oesophagectomy.

† χ^2^ test, except

‡ Mann–Whitney *U* test.

Resection was potentially curative (R0) in 120 (68·2 per cent) after surgery alone *versus* 114 (43·2 per cent) after NACS (*P* = 0·001), and palliative (R1 or R2) in 56 (31·8 per cent) and 150 (56·8 per cent) respectively (*P* < 0·001).

Histopathological examination found CRM involvement in 47 patients (26·7 per cent) after surgery alone and in 112 (42·4 per cent) after NACS (*P* < 0·001). The operative mortality rate (deaths within 30 days) was 4·5 per cent (8 patients) in the surgery‐alone cohort and 2·3 per cent (6 patients) in the NACS cohort (*P* = 0·183).

### Influence of neoadjuvant chemotherapy on disease‐free survival

**Figure 1:**
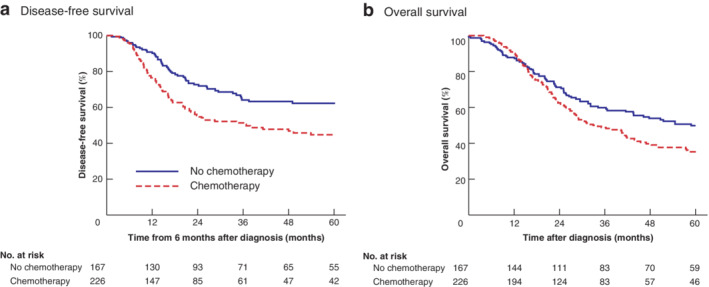
Kaplan–Meier analysis of cumulative disease‐free and overall survival related to treatment modality **a** Disease‐free and **b** overall survival in patients with oesophageal adenocarcinoma who had surgery alone or neoadjuvant chemotherapy followed by surgery. **a** *P* = 0·001, **b** *P* = 0·019 (log rank test).

The relationship between NAC and DFS is shown in *Table* 2 and *Fig*. 1 *a*. NACS was associated with worse 2‐year (50·0 per cent *versus* 70·8 per cent in the surgery‐alone cohort; *P* < 0·001) and 5‐year (32·1 *versus* 56·9 per cent respectively; *P* < 0·001) DFS rates. In univariable analysis, NACS was associated with poorer DFS (HR 1·76, 95 per cent c.i. 1·26 to 2·46; *P* = 0·001). However, in PS analysis, there was no statistical difference between the modalities (*P* = 0·220). The only factor to predict DFS was pTNM stage (HR 3·05, 2·12 to 4·41; *P* < 0·001).

**Table 2:** Univariable and multivariable Cox regression survival analysis of factors influencing disease‐free survival

	Univariable analysis		Multivariable analysis	
	Hazard ratio	*P*	Hazard ratio	*P*
**Preoperative factors**				
Age (< 65 *versus* 66–75 *versus* > 75 years)	1·11 (0·89, 1·37)	0·363		
Sex (F *versus* M)	1·17 (0·75, 1·82)	0·497		
ASA grade (II *versus* III)	0·88 (0·64, 1·21)	0·425		
Neoadjuvant therapy (no *versus* yes)	1·76 (1·26, 2·46)	0·001		0·220
**Operative factors**				
Surgical approach (TTO *versus* THO)	0·92 (0·66, 1·27)	0·599		
pTNM stage (0 *versus* I *versus* II *versus* III *versus* IV)	2·82 (2·21, 3·60)	< 0·001	3·05 (2·12, 4·41)	< 0·001
Differentiation (well/moderate *versus* poor)	2·00 (1·31, 3·03)	0·001		0·611
CRM (negative *versus* positive)	2·35 (1·47, 3·77)	< 0·001		0·454
Lymph node yield (< 15 *versus* ≥ 15)	0·74 (0·53, 1·04)	0·083		0·103
Propensity scores	5·42 (2·85, 10·30)	< 0·001		0·448

Values in parentheses are 95 per cent confidence intervals. TTO, transthoracic oesophagectomy; THO, transhiatal oesophagectomy; CRM, circumferential resection margin.

### Influence of neoadjuvant chemotherapy on overall survival

The relationship between clinicopathological characteristics and OS is shown in *Table* 3 and *Fig*. 1 *b*. NACS was associated with worse 2‐year (48·3 per cent *versus* 64·4 per cent in the surgery‐alone group; *P* < 0·001) and 5‐year (27·5 *versus* 47·3 per cent respectively; *P* < 0·001) OS rates. In univariable analysis, neoadjuvant therapy was associated with poorer OS (HR 1·55, 95 per cent c.i. 1·19 to 2·02; *P* = 0·001). However, in PS analysis there was no statistical difference between the modalities (*P* = 0·431). The only factor to predict OS was pTNM stage (HR 3·27, 2·27 to 4·72; *P* < 0·001).

**Table 3:** Univariable and multivariable Cox regression survival analysis of factors influencing overall survival

	Univariable analysis		Multivariable analysis	
	Hazard ratio	*P*	Hazard ratio	*P*
**Preoperative factors**				
Age (< 65 *versus* 66–75 *versus* > 75 years)	1·16 (0·98, 1·37)	0·092		0·207
Sex (F *versus* M)	1·19 (0·84, 1·68)	0·333		
ASA grade (II *versus* III)	1·23 (0·95, 1·59)	0·122		
Neoadjuvant therapy (no *versus* yes)	1·55 (1·19, 2·02)	0·001		0·431
**Operative factors**				
Surgical approach (TTO *versus* THO)	0·77 (0·58, 1·02)	0·072		0·889
pTNM stage (0 *versus* I *versus* II *versus* III *versus* IV)	2·45 (2·00, 3·00)	< 0·001	3·27 (2·27, 4·72)	< 0·001
Differentiation (well/moderate *versus* poor)	2·22 (1·54, 3·19)	< 0·001		0·125
CRM (negative *versus* positive)	3·15 (2·05, 4·82)	< 0·001		0·655
Lymph node yield (< 15 *versus* ≥ 15)	0·91 (0·69, 1·21)	0·528		
Propensity scores	6·17 (3·48, 10·94)	< 0·001		0·202

Values in parentheses are 95 per cent confidence intervals. TTO, transthoracic oesophagectomy; THO, transhiatal oesophagectomy; CRM, circumferential resection margin.

### Relationship between tumour regression grade and survival

**Figure 2:**
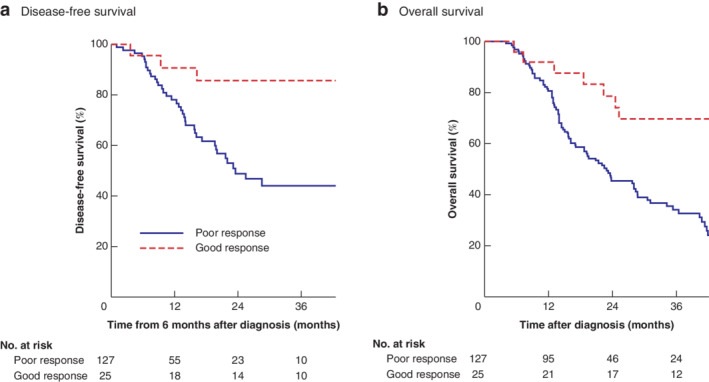
**Kaplan–Meier analysis of cumulative disease‐free and overall survival related to Mandard tumour regression grade** **a** Disease‐free and **b** overall survival in patients with oesophageal adenocarcinoma who had a Mandard tumour regression grade score indicating a good or poor response to neoadjuvant chemotherapy. **a** *P* = 0·005, **b** *P* < 0·001 (log rank test).

Mandard TRG scores, which were recorded routinely in pathology reports, were available for 152 (57·6 per cent) of the 264 patients who had NAC. Twenty‐five patients (16·4 per cent) had a good pathological response (TRG 1–2). Individual Mandard TRG groupings were: TRG 1, 20 patients (13·2 per cent); TRG 2, five (3·3 per cent); TRG 3, 12 (7·9 per cent); TRG 4, 59 (38·8 per cent); and TRG 5, 56 (36·8 per cent). A good Mandard TRG score was associated with improved DFS (*P* = 0·005) (*Fig*. 2 *a*) and OS (*P* < 0·001) (*Fig*. 2 *b*). The 5‐year DFS rate was 64 per cent in the good and 8·0 per cent in the poor Mandard TRG group (*P* < 0·001). Similar findings were observed for OS, with 62 per cent of good responders and 8·6 per cent of poor responders living for 5 years (*P* < 0·001). This equated to a HR of 0·21 (95 per cent c.i. 0·07 to 0·70; *P* = 0·010) for DFS and 0·27 (0·13 to 0·59; *P* = 0·001) for OS.

## Discussion

The principal findings of this study were that, following application of PS adjustment, DFS and OS were comparable for surgery and NAC followed by surgery, both clinically and statistically. Operative morbidity and mortality were higher after surgery alone, but this difference was not statistically significant.

This study has several limitations. It was not a randomized trial, rendering it vulnerable to selection bias and confounding by case mix. Groups of patients were unbalanced in terms of age and stage of disease. Data were from a single regional network. As expected, the process and strategy of radiological staging has developed and improved over the 15 years of the study; CT equipment has advanced and therefore the quality of staging may have been inconsistent. In contrast, the EUS equipment used was not upgraded during the study period, and the implementation of propensity scoring in regression analyses of DFS and OS means much of the selection bias has been considered.

Despite the advantages of PS analysis, the methodology still has limitations, principally the inability to adjust for unknown confounding factors^10^, as well as the assumption that the relationship between the PS and the outcome has been modelled correctly^9^. Consequently, the implementation of PS analysis in observational studies does not negate the use of randomized trials, but rather emphasizes the advantages associated with randomization. In clinical situations where randomization may be impractical, PS analysis is theoretically a way of minimizing bias to obtain results that may approach the level of evidence provided by the rigorous methodology of an RCT^9^, ^23^. PS analysis has two other important strengths. If multivariable model analyses have traditionally been the preferred statistical method for assessing the effect of a predictor variable on outcomes after controlling for baseline characteristics, their appropriateness depends on a consistency with several assumptions underlying any given model. PS analysis has proved to be the most useful statistical method for controlling confounders, providing appropriate estimates even when faced with situations of extreme correlation between the confounders and the exposure^24^. PS analysis is well suited when several risk‐adjusted outcomes are under assessment (DFS and OS), because it simplifies the weighting of multiple outcomes as, once calculated, it can be used for each outcome separately. Allied to PS analysis, this study has additional strengths, in that it is a large study from a regional cancer network, with a well audited practice^16^. Accurate state‐of‐the‐art radiological staging was utilized by means of PET–CT (from 2008) and EUS (all patients)^11^. No patients were lost to follow‐up and dates of death were obtained from the Office for National Statistics, making the survival data especially robust.

A number of RCTs have compared NAC followed by surgery with surgery alone for OAC, but conclusions have differed. The MRC OE02 trial^4^ randomized 802 patients to either treatment arm (9 per cent of patients in each arm also had radiotherapy) and reported significantly improved survival in the NACS arm, with 2‐ and 5‐year survival rates of 34 and 17 per cent after surgery alone, and 43 and 23 per cent after NACS. The RTOG trial 8911^5^, however, reported equivalence, with 2‐ and 5‐year survival rates of 60 and 20·7 per cent after surgery alone, and 59 and 19·4 per cent after NACS, consistent with the present PS analysis. The RTOG trial 8911 did, however, describe a highly significant improvement in survival for patients in the NACS cohort who demonstrated (by way of barium study) a significant response to chemotherapy. Although it was reported^5^ that only 19 per cent of patients in the NACS cohort had major objective disease regression, these patients also received postoperative therapies. Those who did not respond had around a 10 per cent poorer survival than patients who had surgery alone. Response to NAC is heterogeneous, with TRG score correlating with survival, but only around only 16·0 per cent of patients benefit from a good response to NAC, which translates into improved DFS and OS^25^.

The present study has demonstrated similar survival after surgery alone and NAC followed by surgery in patients with OAC, in keeping with the US RTOG trial^5^, but in contrast to the UK MRC OE02 trial^4^. The small subset of patients whose disease responded significantly to NAC (16·4 per cent) nevertheless did have improved survival, compared with those with a poor response. With recent advances in chemotherapy^26^ and the addition of radiotherapy in more recent studies, the best treatment for potentially resectable OAC^27^ remains elusive. For new trials to provide definitive answers, the issue of identifying patients who derive benefit from neoadjuvant therapies, and the development of alternative strategies for those who do not, remains important.

## Collaborators

Members of the South‐East Wales Oesophagogastric Cancer Collaborative include: G. Blackshaw, X. Escofet, A. Foliaki, T. Havard, M. Henwood, J. Witherspoon.

*Disclosure*: The authors declare no conflict of interest.
